# A brief historical perspective of antimicrobial resistance in India

**DOI:** 10.1128/mbio.03548-25

**Published:** 2026-03-27

**Authors:** Maneesh Paul Satyaseela

**Affiliations:** 1 Microvioma Private Limited, Bengaluru, Karnataka, India; Georgia Institute of Technology, Atlanta, Georgia, USA

**Keywords:** AMR, National Action Plan, enmetazobactam, India

## Abstract

Antimicrobial resistance (AMR) is the ability of microbes to survive exposure to antimicrobials that were once effective against them. This is a major global crisis and public health challenge of the 21st century that is declared as a silent pandemic, with India among other countries considered one of the hotspots [R. Laxminarayan, A. Duse, C. Wattal, A. K. M. Zaidi, et al., Lancet Infect Dis 13:1057–1098, 2013, https://doi.org/10.1016/S1473-3099(13)70318-9]. The evolution of AMR in modern India’s context is embedded in a complex interplay of the timing of antibiotic introduction, a growing dense population, cultural inheritances, infectious disease burden, developing health systems, evolving drug market regulations, environmental and animal sector drivers, socioeconomic factors, and policy/implementation gaps. Central to this discussion are the human patient, animal, and environmental aspects of antibiotic overuse from the historical aspects of AMR in India broadly tracing from early antibiotic use to the current “One Health” challenge. It highlights cultural factors, the evolution of surveillance systems, policy responses, India’s efforts, and key lessons in antimicrobial stewardship.

## PERSPECTIVE

## HISTORIC INDIAN WISDOM ON MICROBES AND ANTIMICROBIALS (BC ERA)

Indian medical practices regarding microbes and antimicrobials date back to the evolution of its ancient civilization, but without the current definitions or approach. According to Ayurvedic texts, ancient India’s contributions to bacteriology and microbial pathology emphasize the significance of microorganisms in disease causation and treatment. Herbal remedies, antiseptic metals, and hygiene were part of that civilization, even though there were no modern tools of communication (1, 2). The Indus Valley civilization had advanced sanitation, suggesting hygiene awareness (3). The Atharva Veda mentions Krimi (germs) causing infections, medicinal plants, and treatments, and, finally, Ayurveda (Ayur = life and Veda = science) relies heavily on natural and holistic approaches to keeping the mind, body, and spirit healthy (4). Medicinal plants, for example *Allium sativum*, *Zingiber officinale*, *Camellia sinensis*, *Hypericum perforatum*, *Nigella sativa*, and *Glycyrrhiza glabra*, possess a notable history of efficacy in managing microbial infections (5).

By drawing conceptual links between ancient Vedic healing and contemporary antimicrobial principles that were used under prescription by a Vaidya (practitioner), we can draw parallels by revisiting such traditional knowledge that may offer complementary insights for modern healthcare in India, especially in tackling antimicrobial resistance (AMR) (4).

## THE DAWN OF MODERN ANTIBIOTICS (1940s–1960s)

The discovery of naturally derived antibiotics by Alexander Fleming in 1928 ushered humanity into an era of longevity as these wonder drugs addressed the scourge of infectious diseases. However, this promise of a revolution in public health is short-lived with the emergence of AMR.

In 1949, a penicillin production plant was set up in India as a public-sector undertaking in the drugs and pharmaceuticals sector (6). This coincided almost perfectly with India’s independence in 1947, when one of new India’s priorities was addressing the high burden of infectious diseases. Thus, penicillin and, shortly thereafter, streptomycin and chloramphenicol arrived amid ambitious national campaigns to eradicate major communicable diseases (tuberculosis and typhoid fever) (7). With this intent, initiatives such as establishing public-sector drug units to make antibiotics accessible and affordable to the masses paradoxically fostered a culture of over-reliance on antibiotics among both prescribers and the public. At this time, the concept of resistance, though scientifically known, was unknown to users; thus, stewardship was not considered in the rampant use of antibiotics in humans. By the 1960s, resistance to chloramphenicol had been documented, particularly in Indian hospital settings, but the enthusiasm in antibiotics’ success may have overshadowed such reports at the time (8).

Livestock farming introduced veterinary antibiotics, such as tetracyclines, for the preventive treatment of white scours in calves (9). This heralded the use of antibiotics in animals for prevention, but when it was observed that antibiotic use enhanced animal growth, it was deployed more as a growth promoter.

While many developed countries had an existing tightly controlled drug dispensing system, new India had the priority of taking the reins of a multifaceted nation-building agenda, complexed with a sudden surge in population, and controlling the burden of infectious diseases among others.

## THE RISE OF THE INDIGENOUS PHARMACEUTICAL INDUSTRY (1970s–1990s)

The Indian government’s policy decisions, notably the Drug Price Control Orders, were instrumental in fostering a robust, indigenous pharmaceutical sector. These policies, intended to ensure affordability and self-reliance by limiting prices and foreign company dominance, led to a proliferation of domestic companies specializing in generic drug manufacturing. India became the “pharmacy of the world.” (10) This led to two major unexpected consequences: (i) market saturation with the mass production of a vast array of often irrational, fixed-dose combinations of antimicrobials, leading to competitive cheap prices and, at the same time, many becoming engaged in the manufacture of spurious drugs including antimicrobials; (ii) industrial discharge of antibiotic-laden effluents, creating massive environmental selection pressure for microbial resistance (11). In the 1990s, the arrival of broad-spectrum antibiotics, combined with resistance to older drugs (ampicillin and chloramphenicol), led to a shift toward these new antibiotics, thereby aiding the broadened spectrum of resistance.

## AN ERA OF SATURATION (1990s–2000s)

The self-perpetuating cycle of resistance transmission began across human, animal, and environmental domains, primarily driven by antimicrobial governance systems and infrastructure gaps that were still being scripted. The sheer population density, coupled with socioeconomic realities, amplified the transmission of resistant organisms, clearly reflecting the nexus of these gaps and misuse. This was further fueled by increased over-the-counter (OTC) sales and rampant self-medication, which probably could be due to the generational practice of traditional medicines that were usually self-diagnosed, or quackery (including by the pharmacists), which evolved due to the disproportionate clinician-patient ratio. So, with easy access, people may have adopted antibiotics as part of their self-medication system since the turnaround for community infections was less than a few days. In the meantime, the government started screening for spurious drugs and banning them and introduced Schedule H, followed by Schedule H1, but these did not stop the practice of an OTC culture across the country (12).

The Green Revolution policies and the subsequent rise in animal husbandry added a new layer of selection pressure, with antibiotics used not only for therapeutic purposes but also as preferred growth promoters. This forced human antibiotics such as tetracyclines and fluoroquinolones not only into the food chain but also into the environment (13). This era, with all the above and the booming population, contributed to the next superbug era.

## THE SUPERBUG ERA (2010s TO PRESENT)

The publication of NDM-1 brought sharp focus to the gravity of the situation with infectious diseases that heavily depend on antibiotics (14). The government immediately prioritized its efforts in translating the existing policy framework into a national strategy. The formal policy response began with the development of the National Policy for the Containment of AMR in 2011, followed by the National Action Plan (NAP) on AMR 1.0 in 2017, which aligned with the WHO Global Action Plan (15). This included a critical step: quantifying the problem by establishing the AMR Surveillance and Research Network (AMRSN) by the Indian Council of Medical Research (ICMR). Then came about the regulatory strengthening with the introduction of the Red Line campaign, visually marking antibiotics as prescription-only drugs, which was made stricter with the enforcement of Schedule H1 to curb the OTC culture. One significant step was a ban on colistin as a growth promoter in agriculture, signifying the success of One Health against its misuse. The National Accreditation Board for Hospitals (NABH) initiative helped introduce antimicrobial stewardship programs (ASP) in hospitals, with an emphasis on Infection Prevention and Control (IPC). At the recent G-20, hosted by India, the governments adopted a resolution to address AMR not only in stewardship but also to implement and prioritize tackling it through the One Health approach. It includes research and development (R&D), IPC, and ASP efforts within the respective NAPs, as well as antimicrobial consumption surveillance (16). India’s recent launch of NAP-AMR 2.0 (2025–2029) takes these efforts to the implementation steps in addressing the challenge of AMR in the One Health context with the following 6 strategic objectives: (i) improve awareness and understanding, (ii) strengthen laboratory capacity, (iii) reduce the incidence of infection, (iv) optimize use of antimicrobials, (v) promote research and innovations, and (vi) strengthen governance, coordination, and collaborations (17).

## PROGRESSIVE FUTURE (2025 ONWARD)

India’s NAP-AMR 2.0 marks a significant and timely step forward in addressing the growing threat of AMR, which now extends beyond hospitals and constitutes a true One Health approach. It involves several of the government’s functional ministries with clearer roles and responsibilities, timelines, and a National-State AMR Council for coordination and ensures stronger, more meaningful engagement with the private healthcare and veterinary sectors. NAP-AMR 2.0 also deepens the “One Health” perspective, integrating human, animal, agricultural, food system, and environmental health responses under a unified regulatory framework, strengthening existing strategies and placing greater emphasis on innovation, rapid diagnostics, point-of-care tools, waste management, and improved environmental monitoring.

NAP-AMR 2.0 represents a shift from broad policy intent to a more structured, accountable system that emphasizes actionable and measurable outcomes, with committed leadership, strengthening of state engagement, and cross-sector cooperation. This plan has the potential to translate the strategy into measurable impact in protecting public health and safeguarding antibiotics for future generations. Although the reported AMR statistics for India are highly concerning, the data reported are predominantly from the tertiary care hospitals and do not take into consideration the 1.4 billion population, where AMR prevalence data are lacking.

There are efforts by the government agencies to widen the network to collect the data to have reliable statistics, while in parallel, NAP-AMR 2.0 would aid in curbing the misuse of antibiotics.

## ADDRESSING AMR THROUGH INNOVATION

In addition to these policies and community engagement initiatives, India, known as a generics capital, has now emerged as an innovation hub, especially with enmetazobactam (Exblifep, Orchid Pharma) becoming the first antimicrobial agent invented by India and approved by the United States Food and Drug Administration (USFDA) for the treatment of complicated urinary tract infections, including pyelonephritis, in combination with cefepime. In addition, it is approved by the European Medicines Agency (EMA), the UK Medicines and Healthcare products Regulatory Agency (MHRA), and the Drugs Controller General of India (DCGI) (Orblicef) for the treatment of hospital-acquired, ventilator-associated pneumonia and the associated bacteremia (18). In the advanced invention pipeline from India are Wockhardt’s zidebactam with cefepime (Zaynich), whose NDA was recently accepted by the USFDA (19), and in collaboration between the Indian government and Wockhardt, nafithromycin (Miqnaf) was approved for use in India in Dec 2024 for community-acquired bacterial pneumonia. There are a few promising new chemical entities (NCEs) invented in India (Bugworks and FNDR) among the 90 NCEs in clinical trials of the WHO list (20).

In addition, there are major efforts across the Indian industry, academia, government, and global collaborations to especially develop point-of-care diagnostics for infectious diseases at various stages of development by the ICMR, DBT, CCAMP, FIND, etc. All these demonstrate that India has been making every possible effort to address AMR, especially given the size and diversity of the population, which is significantly diverse and large compared to those of developed countries.

## CONCLUSION

The ancient Indian civilization was rooted in spiritual traditions; these practices parallel modern antibiotic science in their emphasis on the right intervention and prevention. It had many practices passed down through generations, such as cultural and religious practices and home remedies. India is culturally highly diverse, with different kingdoms morphing into modern linguistic states and then becoming a nation; hence, each region poses a significant challenge in harmonious implementation of policies. Unlike many Western nations, where antibiotics were tightly regulated since their introduction, India’s evolving healthcare system and informal medical sector facilitated uncontrolled access, consequently making India’s historical trajectory of AMR unique. The current geopolitical situation presents an excellent opportunity for global health equity partnerships to implement a shift from a dependent system to a practice of self-reliance and sovereignty, termed Viksit Bharat (Developed India).

Following are a few of the reasons to which AMR in independent India can be attributed: (i) population explosion post-Indian independence coinciding with the introduction of modern antibiotics that extended peoples’ quality and length of life; (ii) the nonexistence of antibiotic policy or systematic monitoring when the antibiotics were introduced in the country; (iii) stumbling on the fact that antibiotics not only prevent infections in animals but also improve their growth in farming poultry, animals. and aquaculture; and (iv) the contribution of mass production and export of antibiotics not only to widespread availability domestically but also to environmental contamination through pharmaceutical effluents. Further to this, from an individual perspective, are the following: (v) the self-medication culture driven by incorrect access and affordability, (vi) the cost of consultation and diagnostics versus the cost of buying an antibiotic OTC, (vii) consumers’ pressure to earn without losing a day of sick leave, and (viii) the limited diagnostic infrastructure led to empirical prescribing and a strong “dependence” on the antibiotics by both the prescribers and the patients.

Together, these drivers have created a perfect storm that is being addressed through dynamically evolving policies, regulations, innovations, etc. To bring in any major shift, in my opinion, the priority is in establishing community stewardship to strongly implement the policies and strategies of NAP-AMR 2.0, while experts and governments provide the roadmap and put controls in the supply chain, etc. Unless every citizen, including those authorized to prescribe/dispense antibiotics, becomes aware of and commits to the cause of ASP, the danger of AMR will only get stronger. Hence, there is a strong need to inform the public about the severe consequences of AMR and the importance of adhering to government policies on ASP.

To address AMR, the narrative must now shift to community stewardship along with controlled distribution of antimicrobials by the authorities. NAP-AMR 2.0 can be effective only if everyone imbibes its purpose and practices the guidelines laid out. The main message remains the same: do not use antimicrobials without prescription. Engaging role models (celebrities) or influencers to sensitize people to the severity of the imminent, clear, and present danger posed by the ongoing silent pandemic of AMR is essential. Awareness campaigns among citizens and engagement with organizations such as Rotary International, Lions Club, embassies of various countries, and nongovernmental organizations could give a significant boost to ASP. Microbes evolve through excellent barrier-free “communication” among its species. Similarly, to address AMR with the above commitment to efforts, there needs to be a free flow of education on AMR without barriers. It could be part of the Fit-India initiative of the government. It is essential to have the right communication through the relevant channel to the respective community so that every citizen of the world does their part in preserving the miracle of antimicrobials.

## References

[1] Laxminarayan R, Duse A, Wattal C, Zaidi AKM, Wertheim HFL, Sumpradit N, Vlieghe E, Hara GL, Gould IM, Goossens H, et al.. 2013. Antibiotic resistance-the need for global solutions. Lancet Infect Dis 13:1057–1098. doi:10.1016/S1473-3099(13)70318-9 24252483

[2] Sircar NN. 1991. Ancient Indian bacteriology. Anc Sci Life 10:180–184. 22556529 PMC3331280

[3] Angelakis AN, Capodaglio AG, Passchier CW, Valipour M, Krasilnikoff J, Tzanakakis VA, Sürmelihindi G, Baba A, Kumar R, Haut B, Roubelakis MG, Min Z, Dercas N. 2023. Sustainability of water, sanitation, and hygiene: from prehistoric times to the present times and the future. Water (Basel) 15:1614. doi:10.3390/w15081614

[4] Mahesh S. 2025. Ancient medical knowledge of the atharvaveda and its parallels with modern antibiotic science. RRIJM 10:301–313. doi:10.31305/rrijm.2025.v10.n9.034

[5] Abdallah EM, Alhatlani BY, de Paula Menezes R, Martins CHG. 2023. Back to nature: medicinal plants as promising sources for antibacterial drugs in the post-antibiotic era. Plants (Basel) 12:3077. doi:10.3390/plants12173077 37687324 PMC10490416

[6] UNICEF. 2026. Our history. Available from: https://www.unicef.org/india/our-history

[7] Chakrabarti A, Balaji V, Bansal N, Gopalakrishnan R, Gupta P, Jain A, Kale P, Kapil A, Nath Prasad K, Ray P, Rodrigues C, Walia K. 2025. NAMS task force report on antimicrobial resistance. Ann Natl Acad Med Sci (India) 61:171–209. doi:10.25259/ANAMS_TFR_13_2024

[8] Das B, Chaudhuri S, Srivastava R, Nair GB, Ramamurthy T. 2017. Fostering research into antimicrobial resistance in India. BMJ 6:j3535. doi:10.1136/bmj.j3535 PMC5582344 28877874

[9] Sapre VA, Kulkarni PE. 1970. Observations on the effects of preventive medication with various blendings of “terramycin” (Pfizer) on the incidence of white scours in calves. Indian Vet J 47:694–698.doi:5474636

[10] Kumar S. 2024. Innovative drug discovery research by pharmaceutical companies in India and China. J Med Chem 67:9773–9775. doi:10.1021/acs.jmedchem.4c01180 38857362

[11] Larsson DGJ, de Pedro C, Paxeus N. 2007. Effluent from drug manufactures contains extremely high levels of pharmaceuticals. J Hazard Mater 148:751–755. doi:10.1016/j.jhazmat.2007.07.008 17706342

[12] Kotwani A, Joshi J, Lamkang AS. 2021. Over-the-counter sale of antibiotics in India: a qualitative study of providers’ perspectives across two states. Antibiotics (Basel) 10:1123–1232. doi:10.3390/antibiotics10091123doi:34572705 PMC8472180

[13] Van Boeckel TP, Brower C, Gilbert M, Grenfell BT, Levin SA, Robinson TP, Teillant A, Laxminarayan R. 2015. Global trends in antimicrobial use in food animals. Proc Natl Acad Sci USA 112:5649–5654. doi:10.1073/pnas.1503141112 25792457 PMC4426470

[14] Yong D, Toleman MA, Giske CG, Cho HS, Sundman K, Lee K, Walsh TR. 2009. Characterization of a new metallo-β-Lactamase Gene, bla _NDM-1_, and a novel erythromycin esterase gene carried on a unique genetic structure in Klebsiella pneumoniae sequence type 14 from India. Antimicrob Agents Chemother 53:5046–5054. doi:10.1128/AAC.00774-09 19770275 PMC2786356

[15] Walia K, Madhumathi J, Veeraraghavan B, Chakrabarti A, Kapil A, Ray P, Singh H, Sistla S, Ohri VC. 2019. Establishing antimicrobial resistance surveillance & research network in India: journey so far. Indian J Med Res 149:164–179. doi:10.4103/ijmr.IJMR_226_18doi:31219080 PMC6563732

[16] Ministry of Health and Family Welfare. 2023. Union Health Secretary Chairs Inter-Sectoral Coordination Committee on Anti-Microbial Resistance. Government of India’s press release. https://www.pib.gov.in/PressReleasePage.aspx?PRID=1932681&reg=3&lang=2.

[17] Government of India. 2025. National Action Plan on Antimicrobial Resistance (NAP-AMR) (2025-2029). One Health Approach. https://ncdc.mohfw.gov.in/wp-content/uploads/2025/11/National-Action-Plan-on-Antimicrobial-Resistance-2.0.pdf.

[18] Paul-Satyaseela M. 2025. Invention of enmetazobactam: an indian triumph in antimicrobial drug discovery. ACS Infect Dis 11:1–3. doi:10.1021/acsinfecdis.4c00982doi:39701946

[19] The Economic Times. 2025. Available from: https://economictimes.indiatimes.com/industry/healthcare/biotech/pharmaceuticals/usfda-accepts-wockhardts-nda-for-breakthrough-antibiotic-zaynich/articleshow/125696758.cms?

[20] WHO. 2026. Available from: https://www.who.int/observatories/global-observatory-on-health-research-and-development/monitoring/antibacterial-products-in-clinical-development-for-priority-pathogens

